# PSAT1 regulates hair follicle growth and stem cell behavior in cashmere goats

**DOI:** 10.1186/s12917-025-04736-6

**Published:** 2025-04-16

**Authors:** Xiao-yu Han, Jia-ning Liu, Nan-xiang Sun, Yin-xian Zhang, Hao-bing Bai, Wei-guo Song, Xiao Hu, Hao Liang, Xiong Miao, Yun-mei He, Dong-jun Liu, Xu-dong Guo

**Affiliations:** 1 Key Laboratory of Reproductive Regulation and Breeding of Grassland Livestock, School of Life Sciences, Inner Mongolia University, Hohhot, 010000 China; 2 Inner Mongolia People’s Hospital NHC Key Laboratory of Diagnosis & Treatment of COPD/Inner Mongolia Key Laboratory of Respiratory Diseases, Hohhot, 010000 China; 3 Medical Engineering Department of Inner Mongolia People’s Hospital, Hohhot, 010000 China; 4 Agriculture and Animal Husbandry Technology Extension Center, Etuoke Banner, 016100 China

**Keywords:** Cashmere, Secondary hair follicles, Secondary hair follicle stem cells, Phosphoserine aminotransferase 1, Cell survival, Wound healing, Transcriptomics

## Abstract

**Background:**

The Arbas Cashmere Goat from Inner Mongolia is renowned for its superior-quality cashmere, which is primarily produced by secondary hair follicles (SHFs). Secondary hair follicle stem cells (SHFSCs) are critical regulators of SHF growth and development. However, the specific regulatory mechanisms of phosphoserine aminotransferase 1 (PSAT1) in SHFSCs remain unclear. This study aimed to examine the expression pattern of the PSAT1 gene during SHF cycle transitions in cashmere goats and analyze its effects on SHFSC survival and wound healing.

**Results:**

PSAT1 expression was significantly higher in the anagen phase than in the telogen phase, and was predominantly localized to the bulge region. Functional analyses revealed that elevated PSAT1 expression inhibited SHFSC survival and delayed wound healing; on the other hand, a reduced expression promoted SHFSC survival and accelerated healing. Transcriptomic profiling further demonstrated that PSAT1 expression levels markedly altered the gene expression landscape of SHFSCs. Notably, key signaling pathways essential for hair follicle growth and development, such as Wnt/β-catenin, MAPK, and TGF-β, were significantly affected by PSAT1 modulation.

**Conclusions:**

This study highlights PSAT1 as a critical regulator of SHFSC function in cashmere goats, affecting both cellular survival and regenerative capacity. Through its modulation of multiple signaling pathways, PSAT1 plays a pivotal role in the SHF cycle and may serve as a potential molecular target for improving cashmere fiber production.

**Supplementary Information:**

The online version contains supplementary material available at 10.1186/s12917-025-04736-6.

## Background

Cashmere, a key economic product of the Arbas Cashmere Goat, is globally recognized for its fineness, softness, and warmth [1]. The development of cashmere is a complex process, with its quantity and quality not only being influenced not only by the characteristics of hair follicles (HFs) but also by genetic and environmental factors. The wool and cashmere of cashmere goats consist of medullated fibers produced by primary hair follicles (PHFs) and non-medullated fibers produced by secondary hair follicles (SHFs) [2]. The density, fineness, and yield of cashmere are determined by the quantity and activity of SHFs.

HFs are specialized mini-organs with cyclic growth capabilities [3], comprising the dermal papilla (DP), hair matrix (HM), hair follicle sheath (HS), and bulge [4]. The growth cycle of SHFs in the Arbas Cashmere Goat is divided into the growth phase (anagen), regression phase (catagen), and resting phase (telogen), which occur annually, enabling continuous hair regeneration [5, 6].

Secondary hair follicle stem cells (SHFSCs), located in the bulge region of the outer root sheath of SHFs, are adult stem cells with robust self-renewal capabilities and a multi-lineage differentiation potential; they play a crucial role in SHF growth and development. Although SHFSCs remain mostly quiescent during hair growth, the active proliferation of specific subpopulations contributes to the formation of new hair shafts, a process that is regulated by numerous signaling molecules [7–9].

Phosphoserine aminotransferase 1 (PSAT1) is an enzyme that catalyzes the conversion of phosphoserine to glycine in vivo and plays a significant role in nucleotide synthesis, fatty acid synthesis, and the glutamate cycle [10]. PSAT1 is critical for regulating cell division, proliferation, and energy metabolism, and its dysfunction has been associated with the development of tumors, neurological disorders, and cardiovascular diseases [11]. Furthermore, PSAT1 participates in regulating signaling pathways such as Notch, Wnt/β-catenin, and PI3K-Akt/GSK3β [12, 13], which are closely connected to HF development. However, the role of PSAT1 in the developmental regulation of HFs remains unclear.

In this study, we found that PSAT1 expression was significantly higher during the anagen phase compared to the telogen phase and was primarily localized in the bulge region. By upregulating and downregulating PSAT1 expression in SHFSCs, we demonstrated its impact on SHFSC survival and wound healing. Transcriptomic analysis showed that elevated PSAT1 expression altered signaling pathways related to HF growth and development, including Wnt/β-catenin, MAPK, and TGF-β. These findings provide a theoretical basis for understanding the regulatory mechanisms of HF growth and development and for improving cashmere quality.

## Materials and methods

### Sample collection

Adult Inner Mongolian white cashmere goats (1 year old) were selected from the Yiwei White Cashmere Goat Farm located in Ordos, Inner Mongolia, China. All animals were maintained under standard feeding conditions with unrestricted access to food and water. Skin tissue samples from the dorsal region were collected at two representative stages of the SHF cycle: the telogen phase (April) and the anagen phase (September). At each time point, approximately 1 cm^2^ of full-thickness skin tissue was excised from the sampling site. The tissue samples were immediately fixed in 4% paraformaldehyde and stored at 4 °C for subsequent immunohistochemical analysis.

### Experimental cells and vectors

We utilized the following experimental materials: SHFSCs preserved in our laboratory [14], Escherichia coli Trans100 strain, and pEGFP-C(1) and pcDNA3.1(+) plasmids, which were also stored in our laboratory. These materials were used for the cell transfection and gene expression analyses conducted in this study.

### Cell culture and identification

Arbas Cashmere Goat SHFSCs were cultured in DMEM/F-12 medium (Biological Industries, Kibbutz Beit Haemek, Israel) supplemented with 4% fetal bovine serum (FBS), 14 ng/mL epidermal growth factor (PeproTech, Rocky Hill, NJ, USA), 0.4 µg/mL hydrocortisone (Sigma-Aldrich, Monmouth Junction, NJ, USA), and 10 ng/mL insulin–transferrin–selenium (ITS-X) (Gibco BRL, Grand Island, NY, USA). Cells were maintained in a humidified incubator at 37 °C with 5% CO_2_, and the medium was refreshed every other day. When cells reached 70–80% confluence, experiments were initiated.

### Cell viability assay

The viability of Arbas Cashmere Goat SHFSCs was assessed using a CCK-8 assay kit (Yeasen Biotechnology, Shanghai, China). SHFSCs were seeded into 96-well plates at a density of 4 × 10³ cells per well. Subsequently, 10 µL of CCK-8 reagent was added to each well and was incubated for 2 h at 37 °C in a humidified incubator with 5% CO_2_. Absorbance at 450 nm was measured using a microplate reader to evaluate cell viability. This procedure was repeated daily for 7 consecutive days to generate growth curves. Each experiment was performed in triplicate to ensure reliability.

### PSAT1 gene data acquisition and processing

PSAT1 gene expression data across different HF stages in goats were retrieved from the Ruminant Genome Database (http://animal.omics.pro/code/index.php/main; accessed before November 5, 2024). Data visualization was performed using TBtools software. Additionally, the Gene Expression Library for HFs (https://hair-gel.net; accessed before November 5, 2024) was used to predict PSAT1 expression sites in adult HFs, and visualizations were generated through online services provided by Lian Chuan Bioinformatics (https://www.omicstudio.cn; accessed before November 5, 2024).

### Overexpression vector construction and interference fragment synthesis

The mRNA sequence of the goat PSAT1 gene was retrieved from the NCBI database (https://www.ncbi.nlm.nih.gov/nuccore/XM_005683802.3; accessed before November 5, 2024). Primers for PSAT1 cloning were designed using Primer Premier 5 (Premier Biosoft International, Palo Alto, CA, USA), and the sequences are provided in Supplementary Table S1. Total RNA was extracted from SHFSCs using RNAiso reagent (Takara Bio Inc., Shiga, Japan) and was reverse-transcribed into cDNA using the PrimeScript FAST RT reagent kit with gDNA Eraser (Takara Bio Inc., Shiga, Japan). The cDNA served as a template for subsequent PCR amplification. PCR products were purified using gel recovery and ligated into the pcDNA3.1(+) overexpression vector. The ligation products were transformed into *E. coli* Trans100, and plasmid DNA was extracted and sequenced for verification. The correctly sequenced overexpression vector was named OE-PSAT1.

Interference fragments (si-PSAT1) were synthesized by GenePharma Co., Ltd. (Shanghai, China); the specific sequences are listed in Supplementary Table S2.

### Cell transfection and condition selection

SHFSCs at 70–80% confluence were trypsinized using 0.25% Trypsin-EDTA (Biological Industries, Kibbutz Beit Haemek, Israel) and centrifuged at 1500 rpm for 5 min. After discarding the supernatant, cells were resuspended in Opti-MEM (Gibco BRL, Grand Island, NY, USA) and transferred to electroporation cuvettes. A total of 5 ng of plasmid DNA was added to each cuvette, and the volume was adjusted to 26 µL. Electroporation was carried out using an electroporator (BEX Co., Ltd., Tokyo, Japan) with optimal pulse voltage and time parameters. The resistance range was set between 200 and 600 Ω. To optimize conditions, the pEGFP-C(1) plasmid encoding green fluorescent protein (GFP) was used as a control. GFP expression was observed 48 h post-transfection, and transfection efficiency was quantified using ImageJ software (NIH, Bethesda, MD, USA). Each experiment was repeated three times.

### RT-qPCR

Quantitative real-time PCR (RT-qPCR) was performed using TB Green^®^ Premix Ex Taq™ II (Takara Bio Inc., Shiga, Japan) on the CFX96 Real-Time PCR System (Bio-Rad Laboratories, Hercules, CA, USA). The amplification program included an initial denaturation at 95 °C for 30 s, followed by 40 cycles of 95 °C for 5 s and 60 °C for 34 s. Primers for the target genes were designed using Primer Premier 5 and are listed in Supplementary Table S3. Each reaction was conducted in triplicate, and no-template controls were included. After amplification, a melt curve analysis was performed to confirm product specificity and verify the amplification of a single target.

### Western blotting

Proteins were extracted from processed SHFSCs using the Mammalian Protein Extraction Kit (CWBIO, Beijing, China) following the manufacturer’s protocol. Protein concentrations were subsequently determined using the BCA Protein Assay Kit (Thermo Fisher Scientific, Waltham, WA, USA). Proteins were separated using SDS-PAGE and were transferred onto nitrocellulose membranes. The membranes were blocked at room temperature with 5% non-fat dry milk for 1 h and then incubated overnight at 4 °C with the primary antibody. The following day, the membranes were incubated at room temperature for 1 h with a horseradish peroxidase (HRP)-conjugated secondary antibody. Protein bands were detected using the ECL chemiluminescent substrate (Thermo Fisher Scientific, Waltham, MA, USA). Signals were visualized using the Tanon 5200 imaging system (Tanon, Shanghai, China), and band intensities were quantified with ImageJ software to compare protein expression levels between different groups. Details of the antibodies used are provided in Supplementary Table S4. Each experiment was performed in triplicate.

### Immunofluorescence assay

SHFSCs were seeded evenly into 24-well plates containing cell culture slides (Shanghai Jing An Biological Science and Technology Co., Ltd., Shanghai, China) at a density of 3 × 10⁵ cells per well. When cells reached 60% confluence, they were fixed with 4% paraformaldehyde for 30 min and then permeabilized with 0.5% (v/v) Triton X-100 (Sigma-Aldrich, St. Louis, MO, USA) at room temperature for 10 min. After permeabilization, cells were blocked with 1% bovine serum albumin (BSA) for 30 min. The primary antibody, diluted according to the manufacturer’s recommendations, was applied and incubated overnight at 4 °C. The next day, cells were incubated with an HRP-conjugated secondary antibody at room temperature for 1 h. Finally, cells were stained with 4’,6-diamidino-2-phenylindole (DAPI) for 5 min. Imaging was performed using a confocal laser scanning microscope. Details of the antibodies used are provided in Supplementary Table S5. Each experiment was repeated three times.

### Cell proliferation assay

SHFSCs were seeded evenly into 24-well plates containing cell culture slides at a density of 3 × 10⁵ cells per well. When cells reached 60% confluence, cell proliferation was assessed using the Cell-Light EdU Apollo567 In Vitro Kit (Ribobio, Shanghai, China) according to the manufacturer’s instructions. Imaging was performed using a confocal laser scanning microscope. Each experiment was conducted in triplicate.

### Wound healing assay

SHFSCs were seeded evenly into 6-well plates at a density of 2 × 10⁵ cells per well. Once cells reached 80% confluence, a vertical scratch was made across the cell monolayer using a pipette tip. The wells were rinsed three times with PBS, and fresh culture medium was added. Images were captured under a microscope at 0 h and 48 h, and the cell migration rate was calculated using ImageJ software. Each experiment was repeated three times.

### Flow cytometry

SHFSCs were seeded evenly into 6-well plates at a density of 2 × 10⁵ cells per well. Cells were treated with a Cell Cycle and Apoptosis Detection Kit (7sea Biotech, C001, Shanghai, China) and an Annexin V-FITC/PI Double Staining Apoptosis Detection Kit (7sea Biotech, A005, Shanghai, China). After 48 h, data acquisition was performed using a flow cytometer, and analysis was conducted with FlowJo software (Tree Star, Ashland, OR, USA). Statistical analysis was performed using GraphPad Prism (GraphPad Software, San Diego, CA, USA). Each experiment was repeated three times.

### Transcriptome gene expression analysis

Transcriptome analysis was performed on PSAT1-overexpressing SHFSCs (OE-PSAT1) and control SHFSCs transfected with an empty vector, with three biological replicates per group. Total RNA was extracted using the RNAiso kit (Takara Bio Inc., Shiga, Japan). RNA purity and concentration were assessed using a NanoDrop 2000 spectrophotometer (Thermo Fisher Scientific, Waltham, MA, USA), and RNA integrity was evaluated using an Agilent 5300 Fragment Analyzer System (Agilent Technologies, Santa Clara, CA, USA). The RNA samples exhibited OD260/280 ratios between 1.97 and 2.02 and OD260/230 ratios between 1.98 and 2.22, indicating high purity. The RNA quality was further validated by RQN (RNA Quality Number) scores ranging from 9.4 to 10.0, confirming excellent RNA integrity suitable for transcriptome sequencing. RNA concentrations ranged from 346.4 to 780.9 ng/µL, with total RNA yields between 12.12 and 27.33 µg per sample. The full sample quality data are provided in Supplementary Table S6. Library preparation was conducted using the NEBNext Ultra™ RNA Library Prep Kit for Illumina (New England Biolabs, #E7530L). Sequencing was performed by Shanghai Majorbio Bio-Pharm Technology Co., Ltd. on the Illumina NovaSeq 6000 platform with a paired-end read length of 2 × 150 bp. Each sample produced between 41.4 and 51.6 million raw reads, and after filtering, 40.9–51.0 million clean reads were retained. Clean bases ranged from approximately 6.1 to 7.6 billion per sample. The base error rate was consistently low at 0.0131% across all samples. Quality scores showed high overall sequencing quality, with Q20 values ≥ 98.0% and Q30 values ≥ 94.0%. GC content ranged from 53.95 to 58.22% (Supplementary Table S7). Genome and annotation data for Capra hircus were obtained from the Ensembl database (https://www.ensembl.org/Capra_hircus/Info/Index; accessed before November 5, 2024). Gene expression levels were quantified using the RSEM software package, and detailed expression data are provided in Supplementary Table S8. Differential expression analysis was conducted with DESeq2. Differentially expressed genes (DEGs) were identified based on a fold change (FC) ≥ 2 or ≤ 0.5 and a P-value *<* 0.05. The complete list of DEGs is presented in Supplementary Table S9. Identified DEGs were further used for hierarchical clustering and functional enrichment analyses.

### Statistical analysis

Statistical analyses were performed using GraphPad Prism. For comparisons between two groups, independent sample t-tests were conducted. For comparisons among multiple groups, one-way analysis of variance (ANOVA) followed by Tukey’s multiple comparison test was applied. Results are presented as mean ± standard deviation (mean ± SD), with statistical significance set at *P <* 0.05.

## Results

### PSAT1 displays Stage-Specific and spatially regulated expression patterns

**Fig. 1:**
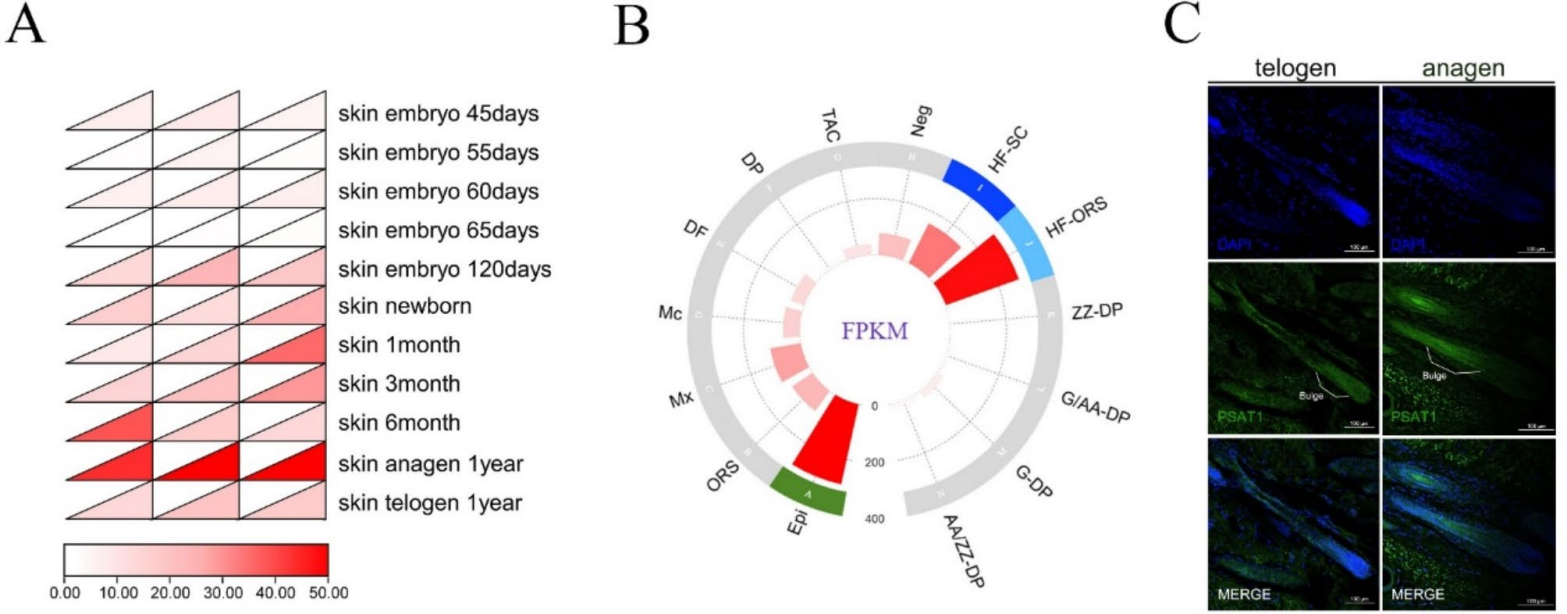
Expression and localization of PSAT1 in cashmere goat HFs. (**A**) PSAT1 expression across the developmental stages of goat HFs. A heatmap displaying the relative expression levels of PSAT1 during the embryonic, adult, anagen, and telogen phases of HFs. (**B**) Predicted expression of PSAT1 in HFs. A bar chart indicating the enrichment of PSAT1 within HF, highlighting higher expression levels in specific regions. (**C**) Immunofluorescence localization of PSAT1 in SHFs. Images showing immunofluorescence staining of PSAT1 in the bulge during the anagen and telogen phases, with cell nuclei counterstained with DAPI (blue). Compared to the telogen phase, the anagen phase bulge exhibits a higher PSAT1 signal intensity. Scale bar = 100 μm

Data retrieved from the Ruminant Genome Database revealed stage-specific and spatially regulated expression patterns of PSAT1 during goat HF development. The analysis indicated that PSAT1 expression was higher in adult HFs compared to those at the embryonic stages, and was also significantly elevated during the anagen phase relative to the telogen phase (Fig. 1A). The prediction of PSAT1 expression sites using the Gene Expression Library for Hair Follicles showed the significant enrichment of PSAT1 in the HF bulge, outer root sheath, and epidermis (Figs. 1B). The immunofluorescence analysis of HF tissue sections further validated these findings, confirming that PSAT1 was primarily expressed in the SHF bulge and exhibited a higher expression during the anagen phase compared to the telogen phase (Fig. 1C). These results aligned with the database predictions.

In summary, the stage-specific and spatially regulated expression patterns of PSAT1 were identified through data analysis, site prediction, and immunofluorescence validation. These findings suggest that PSAT1 plays a significant role in the development and cycling of SHFs.

### PSAT1 regulates the establishment of the microenvironment for SHFSCs

#### Cultivation and characterization of SHFSCs

**Fig. 2:**
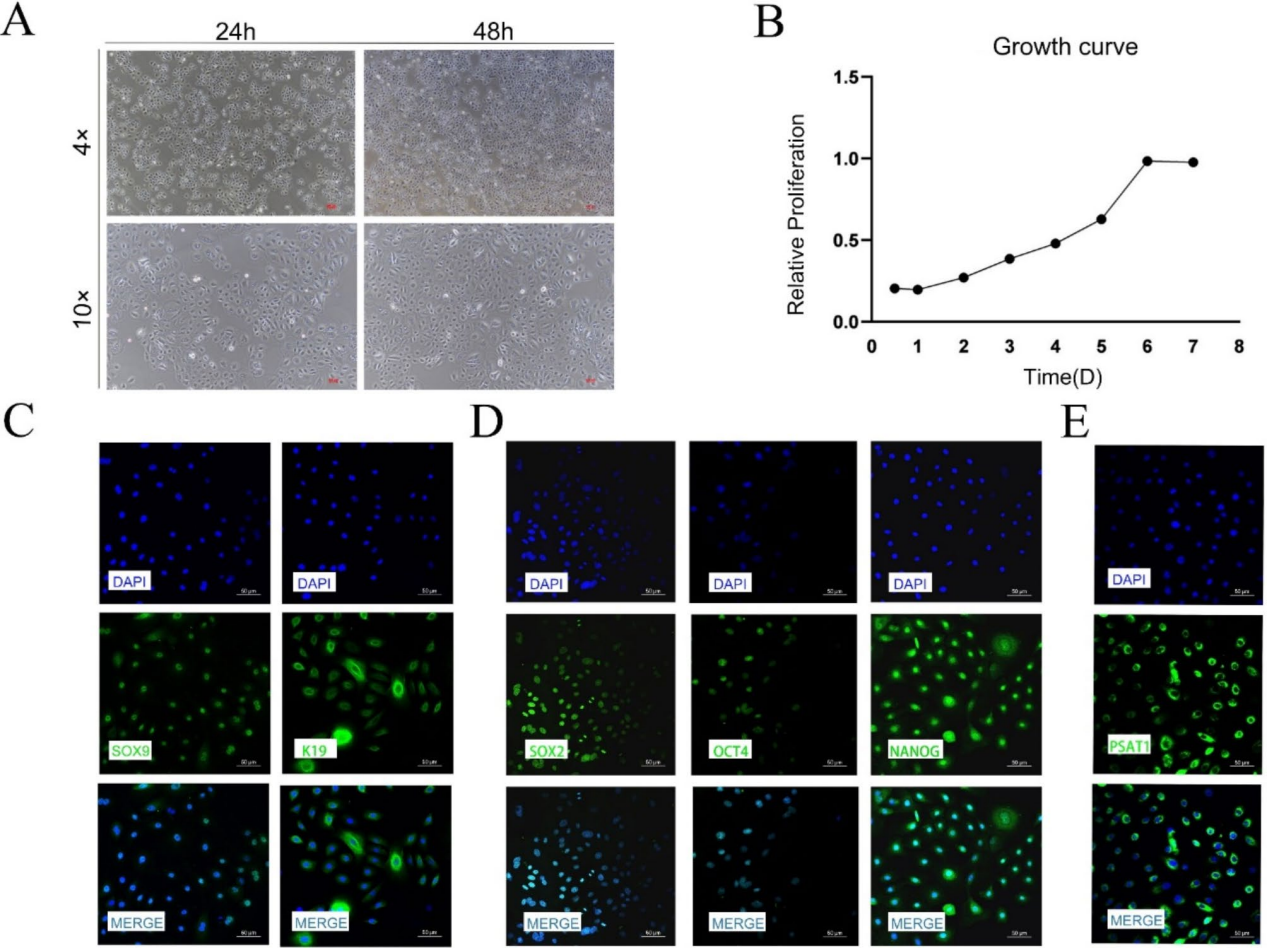
Cultivation and characterization of SHFSCs. (**A**) Cellular morphology and clonal formation. Microscopic images of SHFSCs attached at 24 and 48 h post-seeding, along with the distribution of island-like colonies under 4x and 10x magnification. Scale bar = 100 μm. (**B**) Proliferation curve of SHFSCs. CCK8 assay results depict the proliferation of SHFSCs over time, illustrating a typical sigmoidal growth curve and exponential growth phase. (*n* = 3). (**C**-**E**) Immunofluorescence identification of SHFSCs. Immunofluorescence images demonstrate the identification of SHFSCs using antibodies against SOX9, K19, SOX2, OCT4, NANOG, and PSAT1. Nuclei are stained with DAPI (blue). Scale bar = 50 μm

The post-thaw cultivation of SHFSCs was monitored microscopically (4× and 10× magnification) at 24 and 48 h post-attachment. The cells displayed a smaller volume, droplet-like morphology, good refractivity, and strong adhesion properties, forming island-like clonal distributions. Over time, these clonal areas expanded and merged into larger patches (Fig. 2A).

The proliferation of SHFSCs was analyzed using the CCK-8 assay, which revealed an S-shaped growth curve, indicating vigorous growth. Exponential growth was observed from day 1, with cells entering the logarithmic growth phase on day 6 (Fig. 2B). Immunofluorescence staining was performed to confirm SHFSC identity, using SOX9 and K19 as SHFSC markers and SOX2, OCT4, and NANOG as pluripotency markers. The expression of PSAT1 was also detected in SHFSCs. All tested markers exhibited a positive expression, confirming that the cultured cells were SHFSCs and that PSAT1 was expressed in these cells (Figs. 2C-E).

In summary, SHFSCs exhibited characteristic morphological features and a typical proliferation pattern. Immunofluorescence analysis confirmed the identity of the cells, and PSAT1 expression was successfully detected.

#### Optimization of transfection conditions

**Fig. 3:**
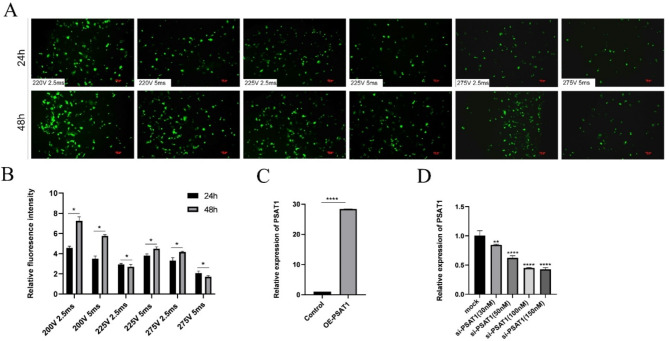
Optimization of transfection conditions. (**A**-**B**) Optimization of electroporation conditions. Fluorescence images compare the transfection efficiency of the pEGFP-C(1) plasmid under various voltage and pulse duration conditions. The bar chart in Figure B displays the fluorescence intensity under each condition. Data are presented as mean ± standard deviation (SD); *n* = 3; *P < 0.05; scale bar = 100 μm. (**C**) Efficiency of PSAT1 overexpression. A bar chart shows the increase in PSAT1 mRNA expression following transfection with the OE-PSAT1 overexpression vector. Data are presented as mean ± SD; *n* = 3; ****P < 0.0001. (**D**) Efficiency of PSAT1 siRNA transfection. A bar chart illustrates the changes in PSAT1 mRNA expression after transfection with different concentrations of si-PSAT1. Data are presented as mean ± SD; *n* = 3; **P < 0.01; ****P < 0.0001

To determine optimal transfection conditions, six different voltage and pulse duration combinations were tested via the electroporation of the pEGFP-C(1) plasmid as a fluorescent reporter (Fig. 3A-B). Fluorescence intensity and cell viability were assessed 48 h post-transfection. The condition of 200 V for 2.5 ms yielded the highest fluorescence intensity and cell viability and was selected for subsequent transfections.

For PSAT1 overexpression, the OE-PSAT1 vector was transfected into SHFSCs. RT-qPCR analysis demonstrated a 28-fold increase in PSAT1 mRNA levels compared to the control group (Fig. 3C). For PSAT1 knockdown, the liposomal transfection of PSAT1 siRNA was optimized by testing five different concentrations (Fig. 3D). Higher siRNA concentrations led to a greater transfection efficiency. At a concentration of 150 nM, the PSAT1 mRNA expression was reduced to 43% of control levels, and this condition was selected for further studies.

In conclusion, optimal transfection parameters for both the overexpression and knockdown of PSAT1 in SHFSCs were established, providing a foundation for further functional studies.

### Elevated PSAT1 expression inhibits SHFSC survival

#### Impact of PSAT1 on SHFSC proliferation

**Fig. 4:**
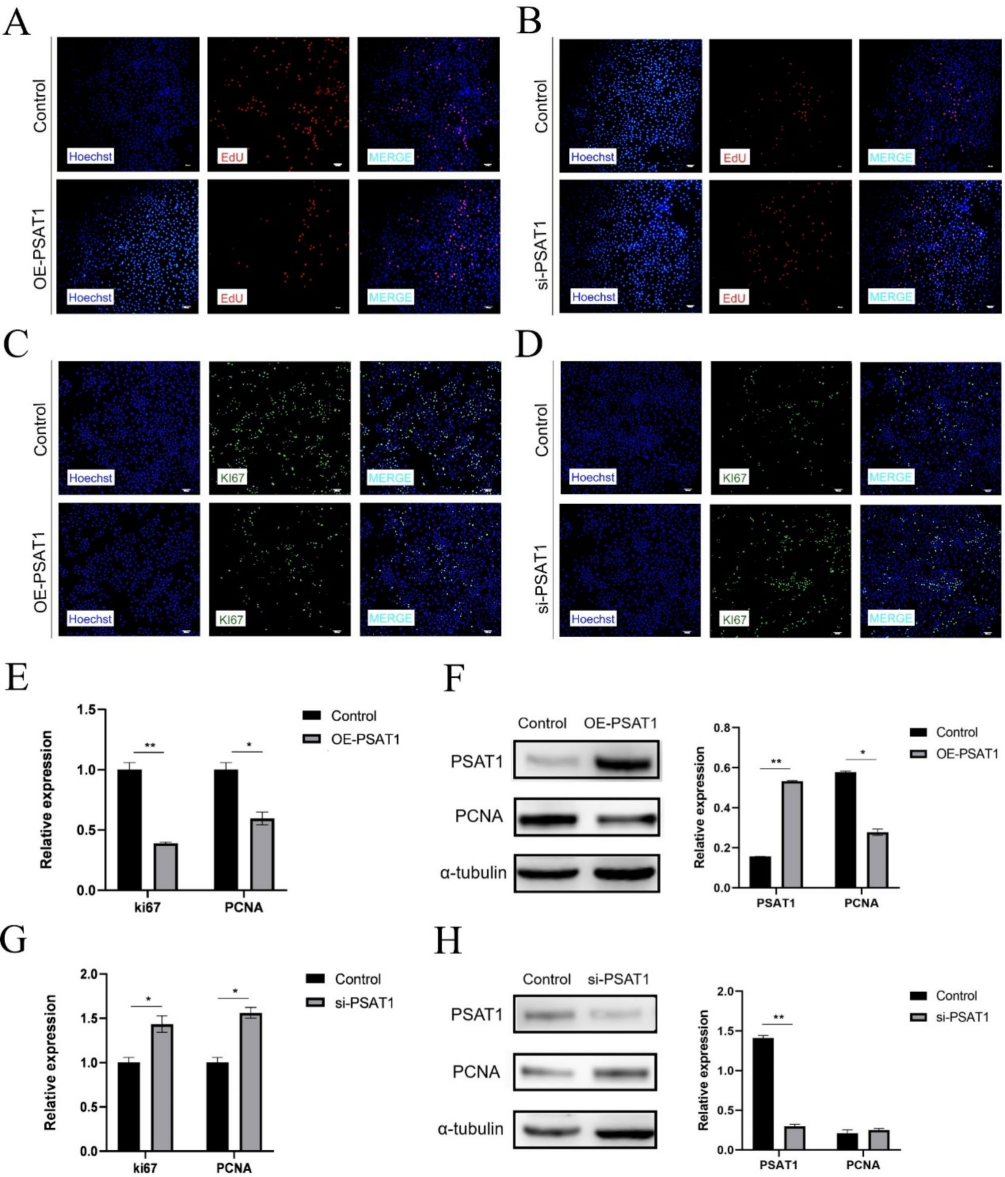
PSAT1 expression levels inhibit SHFSC proliferation. (**A**) EdU positive rate in PSAT1-overexpressing SHFSCs. Compared to the control group, cells overexpressing PSAT1 showed a decreased EdU positive rate. Scale bar = 100 μm. (**B**) EdU positive rate in PSAT1-knockdown SHFSCs. Compared to the control group, cells with PSAT1 knockdown exhibited an increased EdU positive rate. Scale bar = 100 μm. (**C**) Immunofluorescence analysis of Ki67 after PSAT1 overexpression. Images display a weakened fluorescence intensity and a reduced positive cell rate of Ki67 in PSAT1-overexpressing SHFSCs. Scale bar = 100 μm. (**D**) Immunofluorescence analysis of Ki67 after PSAT1 knockdown. Images show an enhanced fluorescence intensity and an increased positive cell rate of Ki67 in PSAT1-knockdown SHFSCs. Scale bar = 100 μm. (**E**) Ki67 and PCNA mRNA expression levels after PSAT1 overexpression. Bar charts indicate that PSAT1 overexpression led to a decrease in Ki67 and PCNA mRNA expression. Data are presented as mean ± standard deviation (SD); *n* = 3; *P < 0.05; **P < 0.01. (**F**) PCNA protein expression levels after PSAT1 overexpression. Western blot analysis shows that PSAT1 overexpression reduced PCNA protein expression. Data are presented as mean ± SD; *n* = 3; *P < 0.05; **P < 0.01. (**G**) Ki67 and PCNA mRNA expression levels after PSAT1 knockdown. Bar charts indicate that PSAT1 knockdown led to an increase in Ki67 and PCNA mRNA expression. Data are presented as mean ± SD; *n* = 3; *P < 0.05. (**H**) PCNA protein expression levels after PSAT1 knockdown. Western blot analysis shows that PSAT1 knockdown increased PCNA protein expression. Data are presented as mean ± SD; *n* = 3; **P < 0.01

To investigate the role of PSAT1 in SHFSC proliferation, EdU assays were conducted on transfected cells. The results demonstrated a decrease in the positive cell rate following PSAT1 overexpression (Fig. 4A), whereas PSAT1 knockdown led to an increase in the positive cell rate (Fig. 4B). Similarly, immunofluorescence assays detecting Ki67 expression revealed a reduced fluorescence intensity and a lower positive cell rate in the PSAT1-overexpression group (Fig. 4C). In contrast, PSAT1 knockdown enhanced fluorescence intensity and increased the positive cell rate (Fig. 4D).

To further analyze the impact of PSAT1 on cell proliferation markers, RT-qPCR and Western blot were performed to assess the mRNA and protein expression levels of Ki67 and PCNA. The overexpression of PSAT1 reduced Ki67 mRNA expression to 0.39-fold and PCNA mRNA expression to 0.60-fold of the control group (Fig. 4E), while PCNA protein expression decreased to 0.48-fold of the control group (Fig. 4F). Conversely, PSAT1 knockdown increased Ki67 mRNA expression to 1.40-fold and PCNA mRNA expression to 1.65-fold of the control group (Fig. 4G). PCNA protein expression also increased to 1.27-fold of the control group (Fig. 4H).

In summary, PSAT1 overexpression inhibited SHFSC proliferation by downregulating Ki67 and PCNA expression, while PSAT1 knockdown promoted proliferation by upregulating these markers. These findings suggest a negative correlation between PSAT1 expression and SHFSC proliferation.

#### Effect of PSAT1 on the cell cycle of SHFSCs

**Fig. 5:**
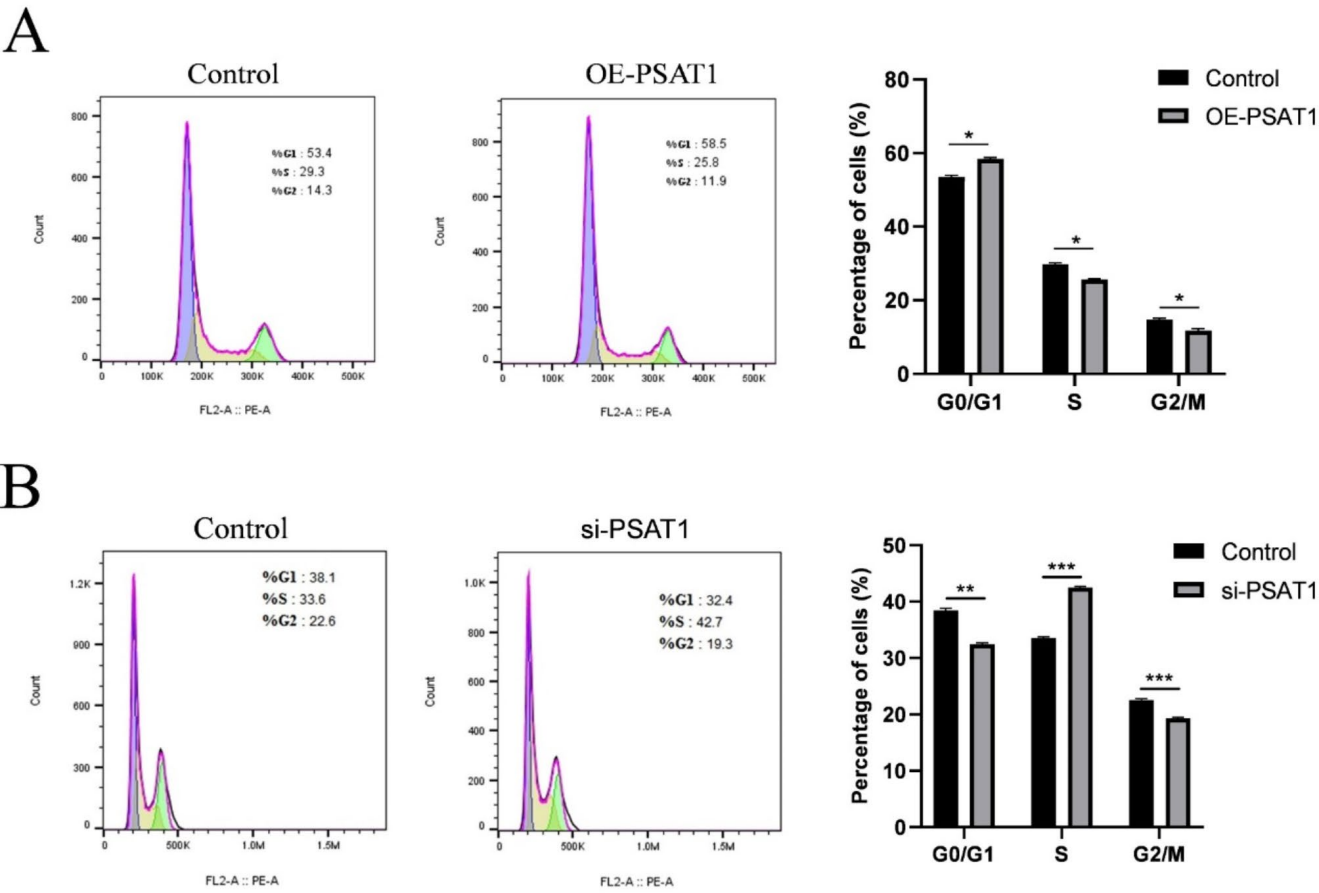
An elevated PSAT1 expression inhibits the cell cycle of SHFSCs. (**A**) The impact of PSAT1 overexpression on the cell cycle of SHFSCs. Flow cytometry analysis indicates that the overexpression of PSAT1 leads to an increase in G0/G1 phase cells and a decrease in S phase and G2/M phase cells. Data are presented as mean ± standard deviation (SD); *n* = 3; *P < 0.05. (**B**) The impact of PSAT1 knockdown on the cell cycle of SHFSCs. Flow cytometry analysis shows that the knockdown of PSAT1 results in a decrease in G0/G1 phase cells, an increase in S phase cells, and a reduction in G2/M phase cells. Data are presented as mean ± standard deviation (SD); *n* = 3; *P < 0.05

Flow cytometry was used to analyze the effects of PSAT1 overexpression and knockdown on the cell cycle of SHFSCs. PSAT1 overexpression increased the percentage of cells in the G0/G1 phase by 5.1%, while decreasing the percentages of cells in the S and G2/M phases by 3.5% and 2.4%, respectively (Fig. 5A). Conversely, PSAT1 knockdown decreased the percentage of cells in the G0/G1 phase by 5.7%, increased the percentage of cells in the S phase by 9.1%, and decreased the percentage.

of cells in the G2/M phase by 3.3% (Fig. 5B).

In conclusion, PSAT1 overexpression resulted in cell cycle arrest at the G0/G1 phase, inhibiting cell cycle progression, while PSAT1 knockdown promoted the transition of cells from the G0/G1 phase to the S phase. These results suggest that PSAT1 negatively regulates the cell cycle of SHFSCs.

#### Impact of PSAT1 on SHFSC apoptosis

**Fig. 6:**
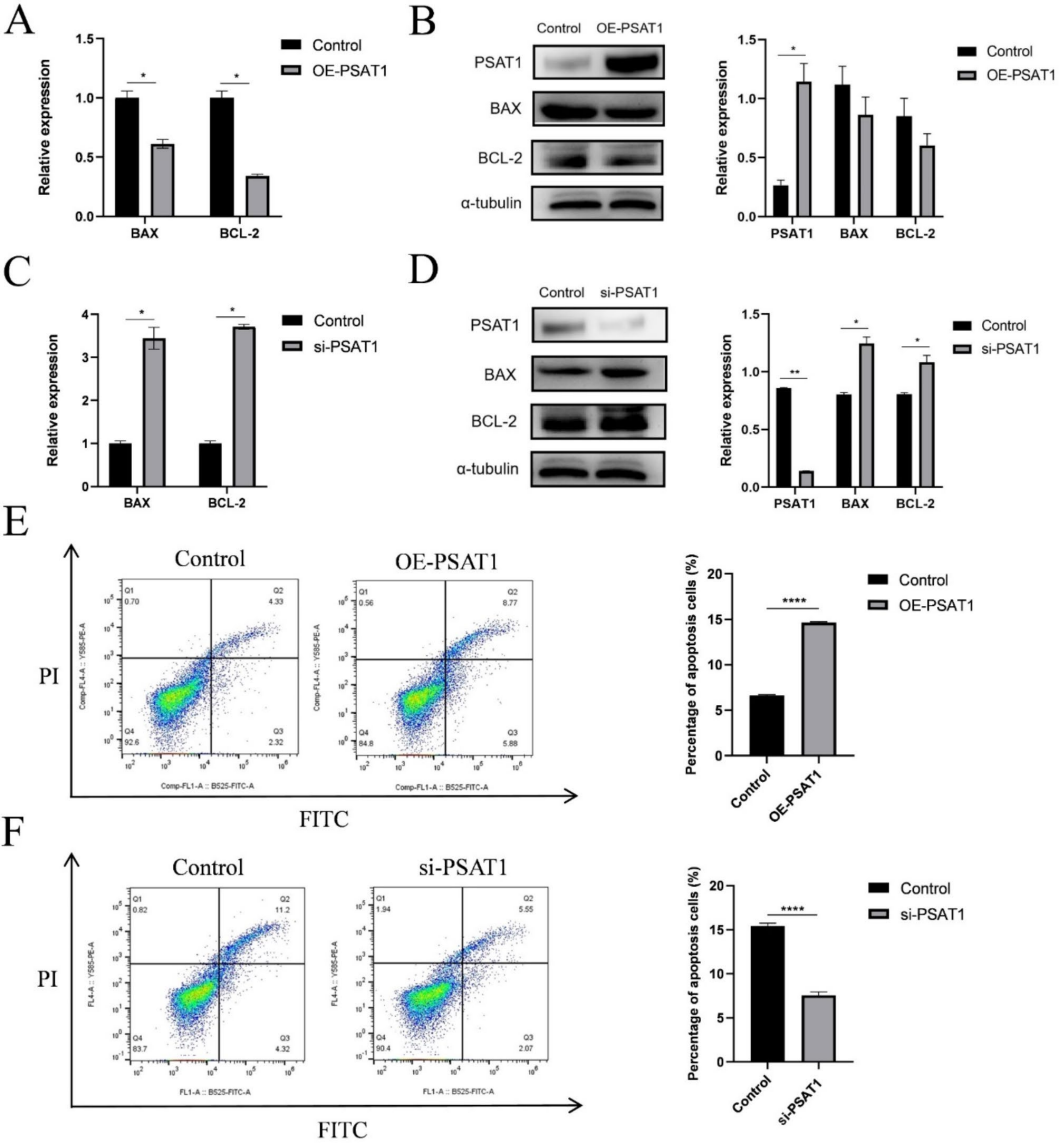
PSAT1 expression levels promote SHFSC apoptosis. (**A**) BCL-2 and BAX mRNA expression levels after PSAT1 overexpression. Bar graphs show decreased mRNA expression levels of BCL-2 and BAX, as well as a reduced BCL-2/BAX ratio in cells overexpressing PSAT1 compared to the control group. Data are presented as mean ± standard deviation (mean ± SD); *n* = 3; *P < 0.05. (**B**) BAX and BCL-2 protein expression levels after PSAT1 overexpression. Western blot analysis indicates a decrease in BAX and BCL-2 protein expression relative to the control group following PSAT1 overexpression. Data are presented as mean ± SD; *n* = 3; *P < 0.05. (**C**) BCL-2 and BAX mRNA expression levels after PSAT1 knockdown. Bar graphs show increased mRNA expression levels of BCL-2 and BAX, as well as an elevated BCL-2/BAX ratio in cells with PSAT1 knockdown compared to the control group. Data are presented as mean ± SD; *n* = 3; *P < 0.05. (**D**) BAX and BCL-2 protein expression levels after PSAT1 knockdown. Western blot analysis indicates an increase in BAX and BCL-2 protein expression relative to the control group following PSAT1 knockdown. Data are presented as mean ± SD; *n* = 3; *P < 0.05; **P < 0.01. (**E**) Apoptosis rate after PSAT1 overexpression. Annexin V-FITC/PI double staining and flow cytometry analysis reveal an increase in the apoptosis rate of cells with PSAT1 overexpression compared to the control group. Data are presented as mean ± SD; *n* = 3; ****P < 0.0001. (**F**) Apoptosis rate after PSAT1 knockdown. Annexin V-FITC/PI double staining and flow cytometry analysis reveal a decrease in the apoptosis rate of cells with PSAT1 knockdown compared to the control group. Data are presented as mean ± SD; *n* = 3; ****P < 0.0001

The impact of PSAT1 on SHFSC apoptosis was evaluated by measuring the mRNA and protein expression levels of BAX and BCL-2, which are key regulators of apoptosis. At the mRNA level, PSAT1 overexpression reduced BCL-2 and BAX expression to 0.36-fold and 0.58-fold of the control group, respectively, leading to a reduction in the BCL-2/BAX ratio to 0.62-fold (Fig. 6A). In contrast, PSAT1 knockdown increased BCL-2 and BAX expression to 3.71-fold and 3.38-fold of the control group, respectively, and elevated the BCL-2/BAX ratio to 1.10-fold (Fig. 6C).

At the protein level, PSAT1 overexpression decreased the expression of BCL-2 and BAX to 0.71-fold and 0.77-fold of the control group, respectively (Fig. 6B). Conversely, PSAT1 knockdown increased BCL-2 and BAX protein expression to 1.35-fold and 1.56-fold of the control group, respectively (Fig. 6D). Annexin V-FITC/PI double staining and flow cytometry further confirmed these findings, revealing that PSAT1 overexpression increased the apoptosis rate by 8% (Fig. 6E), whereas PSAT1 knockdown decreased the apoptosis rate by 7.9% (Fig. 6F).

In summary, these results demonstrate that PSAT1 is positively correlated with SHFSC apoptosis. The overexpression of PSAT1 promotes apoptosis, whereas *PSAT1* knockdown inhibits apoptosis.

### Elevated expression levels of PSAT1 impair the wound healing capacity of SHFSCs

**Fig. 7:**
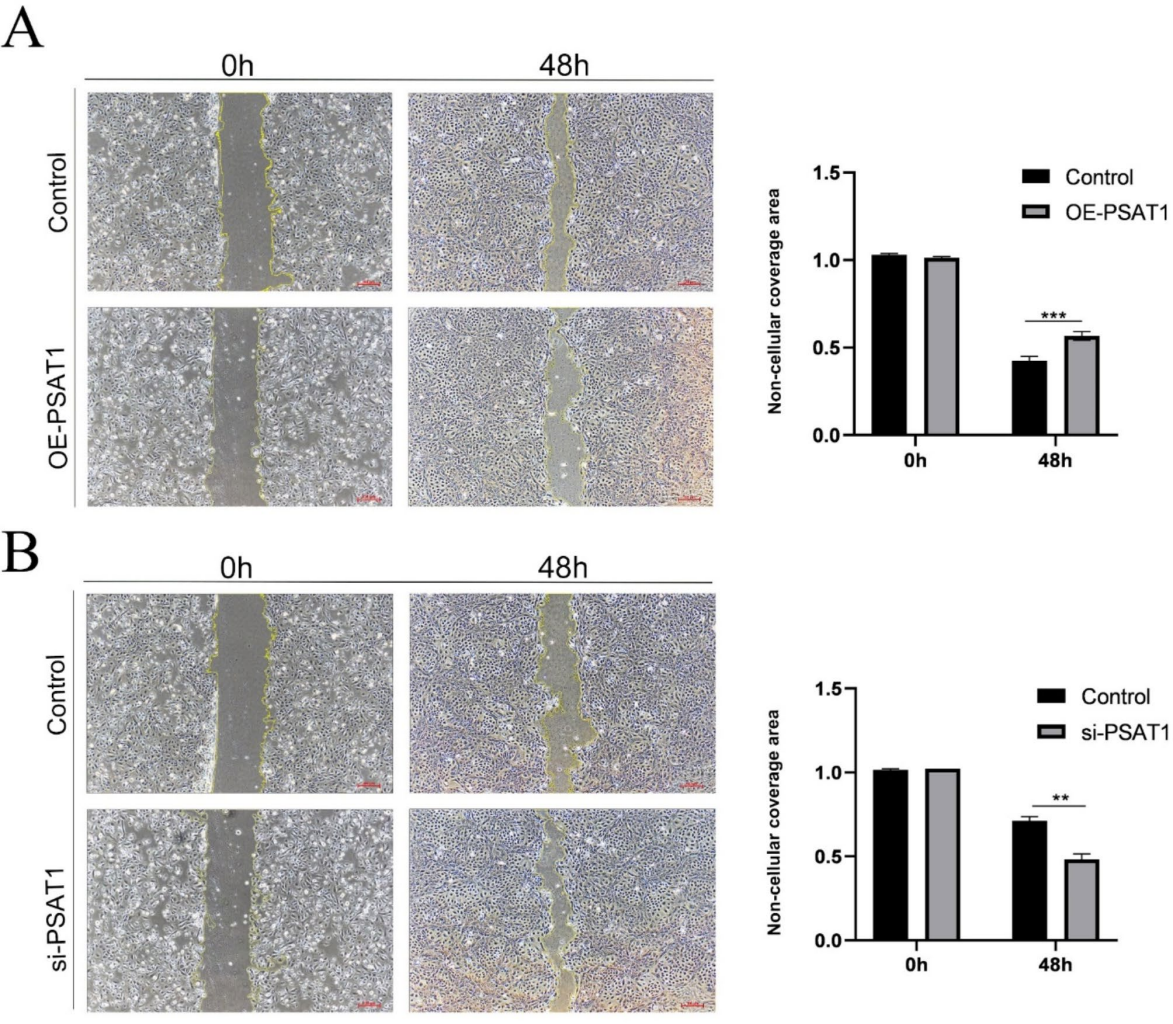
Elevated PSAT1 expression decreases SHFSC migration ability. (**A**) Wound healing assay after PSAT1 overexpression. Images demonstrate a reduced wound healing rate in PSAT1-overexpressing SHFSCs compared to the control group. Scale bar = 100 μm. Data are presented as mean ± standard deviation (mean ± SD); *n* = 3; **P < 0.001. (**B**) Wound healing assay after PSAT1 knockdown. Images show an accelerated wound healing rate in PSAT1-knockdown SHFSCs compared to the control group. Scale bar = 100 μm. Data are presented as mean ± standard deviation (mean ± SD); *n* = 3; **P < 0.01

To examine the effect of PSAT1 on the migratory ability of SHFSCs, wound healing assays were performed to measure the area of cell confluence 48 h post-wounding. The results showed that the scratch wound healing rate was significantly reduced in SHFSCs with PSAT1 overexpression, whereas PSAT1 knockdown enhanced the healing rate (Fig. 7A-B).

In summary, these findings suggest that PSAT1 negatively regulates the migratory capacity of SHFSCs. The overexpression of PSAT1 impairs migration, while its knockdown enhances this ability, indicating a negative correlation between PSAT1 expression and SHFSC migration.

### PSAT1 alters the transcriptome of SHFSCs

#### PSAT1’s impact on the SHFSC transcriptome

**Fig. 8:**
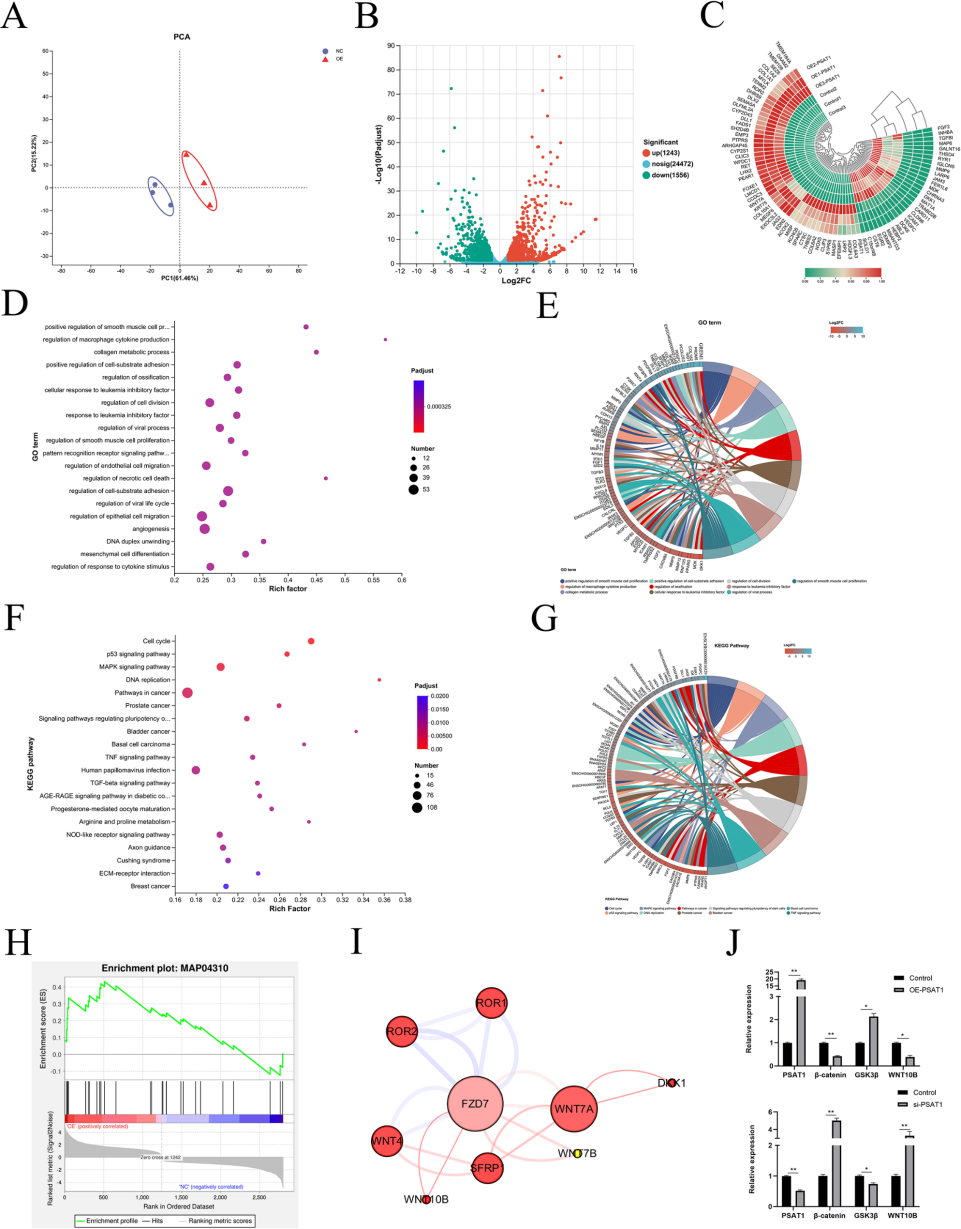
PSAT1 alters the transcriptome of SHFSCs. (**A**) Principal component analysis (PCA) of samples. The PCA demonstrates the differential gene expression and sample reproducibility between the experimental group (OE-PSAT1) and the control group (Control). (*n* = 3). (**B**) Volcano plot of differentially expressed genes (DEGs). The plot displays upregulated (red) and downregulated (green) DEGs, along with their quantities (1243 upregulated, 1556 downregulated). FC ≥ 2 or FC ≤ 0.5; *P < 0.05. (**C**) Hierarchical clustering analysis of DEGs. A heatmap illustrating the expression patterns of the top 100 significantly differentially expressed genes between the experimental and control groups. The scale bar represents normalized expression values. (**D**) GO enrichment analysis of DEGs. A bubble chart showing the top 20 significantly enriched GO terms, primarily involving biological functions such as cell proliferation, migration, and metabolic process regulation. (**E**) GO enrichment string plot for hair-follicle-development-related genes. The plot highlights genes related to HF development, including SFRP1, BMP4, WNT10B, TGFβ2, and WNT4. (**F**) KEGG enrichment analysis of DEGs. A bubble chart displaying the top 20 significantly enriched KEGG pathways, including cancer pathways, the MAPK signaling pathway, and the TGF-β signaling pathway. (**G**) KEGG enrichment string plot for hair-follicle-development-related genes. The plot showcases genes significantly related to HF development, such as WNT7A, WNT4A, FZD7, LEF1, and WNT10B. (**H**) GSEA of DEGs. The chart indicates a significant enrichment of the Wnt signaling pathway, with the positive correlation line (red) rapidly ascending after the zero-crossing point, suggesting a positive correlation with cell survival. (**I**) Protein–protein interaction analysis of sig-nificantly enriched key factors in the Wnt signaling pathway. The analysis shows a significant enrichment of genes such as Wnt7A, Wnt10B, and FZD7. (**J**) RT-qPCR validation of transcriptome data. Bar graphs display the mRNA expression changes in PSAT1, β-catenin, GSK3β, and WNT10B genes following PSAT1 overexpression and knockdown, consistent with the results of transcriptome sequencing. Data are presented as mean ± standard deviation (mean ± SD); *n* = 3; *P < 0.05; **P < 0.01

To investigate the effects of PSAT1 on the transcriptome of SHFSCs, high-throughput RNA sequencing was performed on PSAT1-overexpressing SHFSCs (OE-PSAT1) and control SHFSCs transfected with an empty vector (control), with three biological replicates in each group. Following quality control and sequence alignment, 16,103 expressed genes were identified. Principal component analysis (PCA) revealed significant differences in gene expression between the experimental and control groups, with a high intra-group repeatability, confirming the reliability of the experimental procedures (Fig. 8A).

A quantitative analysis of gene expression identified differentially expressed genes (DEGs) using a cutoff of fold change (FC) ≥ 2 or FC ≤ 0.5 and a significance level of *P <* 0.05. A total of 2,799 DEGs were detected, including 1,243 upregulated and 1,556 downregulated genes in the experimental group compared to the control (Fig. 8B). A cluster analysis of DEGs showed consistent expression patterns within replicates from both groups, further validating the stability of the experimental data (Fig. 8C).

#### Biological function of DEGs

To understand the biological roles of DEGs, Gene Ontology (GO) enrichment analysis was performed. The results showed that DEGs were primarily enriched in biological processes related to cell proliferation, migration, and metabolic regulation (Fig. 8D). GO enrichment string analysis further identified key genes associated with HF development, including SFRP1, BMP4, WNT10B, TGFβ2, and WNT4 (Fig. 8E).

Kyoto Encyclopedia of Genes and Genomes (KEGG) pathway analysis revealed that DEGs were involved in pathways strongly associated with HF development. These included cancer-related pathways, as well as the MAPK signaling, TGF-β signaling, Wnt/β-catenin signaling, Hedgehog signaling, and BMP signaling pathways (Fig. 8F). KEGG enrichment string analysis highlighted specific genes such as WNT7A, WNT4A, FZD7, LEF1, and WNT10B, which are significantly related to HF development (Fig. 8G). Gene Set Enrichment Analysis (GSEA) showed a significant enrichment of the Wnt signaling pathway, indicating that genes within this pathway ranked high in the dataset and were positively correlated with cell survival (Fig. 8H). Protein–protein interaction (PPI) analysis further revealed a significant enrichment of key factors in the Wnt signaling pathway, with WNT7A, WNT10B, and FZD7 being identified as critical genes involved in HF development (Fig. 8I).

To validate the RNA sequencing data, RT-qPCR was performed on six key DEGs. The results confirmed that PSAT1 overexpression elevated its own mRNA expression to 19.2-fold that of the control group, while reducing β-catenin expression to 0.41-fold, increasing GSK3β expression to 2.13-fold, and decreasing WNT10B expression to 0.38-fold of control levels. Conversely, following PSAT1 knockdown, PSAT1 mRNA expression was reduced to 0.42-fold, β-catenin expression increased to 5-fold, GSK3β expression decreased to 0.74-fold, and WNT10B expression increased to 3.28-fold compared to controls (Fig. 8J). These results were consistent with the RNA sequencing data, confirming the reliability of the transcriptomic analysis.

In conclusion, high-throughput transcriptome sequencing demonstrated a significant role for PSAT1 in regulating the transcriptome of SHFSCs. Changes in PSAT1 expression were closely associated with alterations in the expression of key genes involved in cell proliferation, migration, and metabolic processes, as well as with the modulation of multiple signaling pathways critical for HF development.

## Discussion

The HF growth cycle consists of the anagen, catagen, and telogen phases, forming a continuous process of hair growth, shedding, and regeneration [11, 15]. SHFSCs, located in the bulge region of the HF, possess the ability to self-renew and differentiate into multiple cell types. Our study demonstrates that changes in PSAT1 expression are closely associated with this cycle, particularly in cell proliferation, apoptosis, and migration. Specifically, PSAT1 overexpression inhibits the proliferation and migration of SHFSCs, whereas PSAT1 knockdown promotes these processes. These findings align with previous studies that identified PSAT1 as a regulator of the cell cycle and migration in various cell types [16, 17], highlighting its key role in HF growth cycle regulation.

To gain mechanistic insight into the observed phenotypic changes, we performed transcriptomic profiling of SHFSCs following PSAT1 overexpression. The analysis revealed that PSAT1 overexpression significantly altered the expression of genes involved in cell proliferation, cell cycle regulation, apoptosis, and migration, which is consistent with our in vitro functional assays.

For example, genes that negatively regulate the cell cycle, such as CDKN1A and CCNG2, were significantly upregulated, while positive regulators including PLK1 and CDK2 were downregulated (Supplementary Table S9). These changes likely contribute to the observed G0/G1 phase arrest and reduced cell proliferation [18–20]. In terms of apoptosis, pro-apoptotic genes such as CASP6 were upregulated, whereas anti-apoptotic genes like BCL2 were markedly downregulated, correlating with the increased apoptotic rates in PSAT1-overexpressing SHFSCs [21, 22].

In addition, several genes associated with cell migration and extracellular matrix remodeling exhibited significant changes. MMP1, ITGB4, and VIM, which are critical for cell motility and adhesion, were significantly downregulated, consistent with the impaired migratory capacity observed in the wound healing assay [23, 24]. These transcriptomic findings support the hypothesis that PSAT1 regulates SHFSC behavior through the coordination of multiple gene networks.

KEGG enrichment analysis further identified PSAT1’s involvement in regulating the Wnt/β-catenin, MAPK, and TGF-β signaling pathways, all of which are crucial for HF development and growth [25–27]. These findings are consistent with recent studies that highlight PSAT1’s role in modulating cellular signal transduction [28, 29].

Among these, Wnt/β-catenin signaling appears to be particularly relevant. β-catenin, a central mediator of this pathway, functions by precisely controlling its cytoplasmic levels [30, 31]. Activated Wnt signals inhibit GSK3β activity, leading to β-catenin accumulation and nuclear translocation, where it partners with TCF/LEF family transcription factors to regulate gene expression. WNT ligands such as WNT1, WNT3A, WNT7A, and WNT10B are well-established activators of this cascade [32, 33]. Importantly, this pathway interacts with others, including TGF-β/BMP, to jointly regulate HF stem cell fate [34–36].

Our RT-qPCR results further confirmed that PSAT1 overexpression decreases the mRNA expression of β-catenin and WNT10B, while increasing GSK3β expression. Conversely, PSAT1 knockdown led to upregulation of β-catenin and WNT10B and downregulation of GSK3β. These results suggest that PSAT1 modulates SHFSC proliferation and differentiation, at least in part, through its impact on the Wnt/β-catenin pathway.

Interestingly, the function of PSAT1 in SHFSCs appears to parallel its role in tumor biology. Previous studies have shown that PSAT1 influences cancer cell behavior by modulating cell cycle progression and apoptotic pathways, as well as contributing to metabolic reprogramming [37–39]. These findings suggest that PSAT1 may exert conserved regulatory functions across distinct cellular contexts, including stem cell maintenance, tissue regeneration, and tumor development.

Despite the strengths of this study, several limitations should be acknowledged. First, the transcriptomic analysis was conducted in vitro using overexpression and knockdown models, which may not fully reflect the complexity of the in vivo HF microenvironment. Second, although KEGG analysis identified key pathways such as Wnt and MAPK, further functional experiments—such as pathway inhibition, rescue assays, or in vivo validation—are necessary to confirm causal relationships. Third, the RNA-Seq sample size was relatively small (*n* = 3 per group), which may limit generalizability. Additionally, the current study focused on short-term effects of PSAT1 modulation; its long-term impact across different HF cycle stages remains to be elucidated.

Future work should incorporate time-course studies, lineage tracing, and in vivo models to clarify PSAT1’s role in hair follicle biology and determine whether it can serve as a therapeutic target or biomarker for hair regeneration.

In conclusion, this study highlights the critical role of PSAT1 in regulating the growth characteristics of SHFSCs and identifies the signaling pathways it modulates. Our integrative transcriptomic and functional analysis provides a mechanistic foundation for understanding the molecular basis of PSAT1-mediated regulation in the HF niche, offering new insights for improving cashmere quality and HF regeneration strategies.

## Conclusions

This study successfully revealed the differential expression of PSAT1 during the HF cycle in cashmere goats and its regulatory role in the survival and migration of SHFSCs. Our results demonstrated that PSAT1 expression was significantly higher during the anagen phase compared to the telogen phase, and its expression levels directly influenced the proliferation and apoptosis of SHFSCs. Furthermore, the regulatory effects of PSAT1 on key signaling pathways, including Wnt/β-catenin, MAPK, and TGF-β, were shown to play critical roles in HF growth and development.

## Electronic supplementary material

Below is the link to the electronic supplementary material.

Supplementary Material 1

Supplementary Material 2

Supplementary Material 3

Supplementary Material 4

Supplementary Material 5

Supplementary Material 6

Supplementary Material 7

Supplementary Material 8

Supplementary Material 9

## Data Availability

The datasets generated and analyzed during the current study are available in the NCBI Sequence Read Archive (SRA) under the accession number PRJNA1209128.
