# Supplementary material for: PSAT1 regulates hair follicle growth and stem cell behavior in cashmere goats
Source: BMC Vet Res. 2025 Apr 16;21:277. doi: 10.1186/s12917-025-04736-6 (PMC12001598; doi:10.1186/s12917-025-04736-6)
Supplement: Supplementary file 1 — Supplementary Material 1 [file 12917_2025_4736_MOESM1_ESM.pdf]

Supplementary Table S1. PCR Primer Information

Primer Name	Sequence (5'-3')	Length (bp)
PSAT1-F	GGACTCCCTGATACCG	1113
PSAT1-R	AACCTTTCTTCTACCCACT	
