# Supplementary material for: PSAT1 regulates hair follicle growth and stem cell behavior in cashmere goats
Source: BMC Vet Res. 2025 Apr 16;21:277. doi: 10.1186/s12917-025-04736-6 (PMC12001598; doi:10.1186/s12917-025-04736-6)
Supplement: Supplementary file 2 — Supplementary Material 2 [file 12917_2025_4736_MOESM2_ESM.pdf]

Supplementary Table S2. siRNA Interference Fragment Information

siRNA Name	Sequence (5'-3')	Target Gene	Length (bp)
Sense Oligo	GGGAUUAGUGUUCU UGAAATT	PSAT1-goat-221	21
Anti-sense Oligo	UUUCAAGAACACUA AUCCCTT		21
