# Supplementary material for: PSAT1 regulates hair follicle growth and stem cell behavior in cashmere goats
Source: BMC Vet Res. 2025 Apr 16;21:277. doi: 10.1186/s12917-025-04736-6 (PMC12001598; doi:10.1186/s12917-025-04736-6)
Supplement: Supplementary file 3 — Supplementary Material 3 [file 12917_2025_4736_MOESM3_ESM.pdf]

Supplementary Table S3. RT-qPCR Primer Information

Gene Name	Sequence (5'-3')	Length (bp)
GAPDH	F: TTCCACGGCACAGTCAAGG<br>R: CTCAGCACCAGCATCACCC	114
PSAT1	F: GAAGCCAAGAAGTTCGG<br>R: CATCAGGGATAAAGTCAAA	158
Ki67	F:CCCAGTATTAAAATGAGCG<br>R:TGATGTTGCCTTTGGAGTC	92
PCNA	F:CTTGAAGAAAGTGCTGGAG<br>R:TGGACATGCTGGTGAGG	93
BCL-2	F: TGTGGATGACCGAGTACCTGAA<br>R:AGACAGCCAGGAGAAATCAAACA	120
BAX	F:TCCGACGGCAACTTCAA<br>R:ACAGGGACAGCAGGCAC	96
β-catenin	F: AGGATACCCAGCGTCGTA<br>R: TGTGAAGGGCTCCAGTA	102
GSK3β	F: TAACATAGTCCGATTGCG<br>R: ATCACAGGGAGCGTCT	147
WNT7A	F: TTCGGCGGGGATGGTGT<br>R:ACTTGAGGTGCATGTGACTGG	127
WNT10B	F: ATCGCGGTCCACGAGTGT<br>R: AGCCAGCATGGAGAAGGAAA	144
