# Supplementary material for: PSAT1 regulates hair follicle growth and stem cell behavior in cashmere goats
Source: BMC Vet Res. 2025 Apr 16;21:277. doi: 10.1186/s12917-025-04736-6 (PMC12001598; doi:10.1186/s12917-025-04736-6)
Supplement: Supplementary file 4 — Supplementary Material 4 [file 12917_2025_4736_MOESM4_ESM.pdf]

Supplementary Table S4. Information of Antibodies Used in This Study for WB Assays.

**Antibodies**	**Source**	**Identifier**	**Host**	**Proportion**
Anti -PCNA	Proteintech	10205-2-AP	Rabbit	1:10000
Anti -BAX	Proteintech	50599-2-IG	Rabbit	1:5000
Anti -BCL2	Proteintech	12789-1-AP	Rabbit	1:5000
Anti -PSAT1	Proteintech	10501-1-AP	Rabbit	1:10000
Anti -alpha Tubulin	Abcam	ab7291	Mouse	1:7500
Anti - Rabbit IgG	Proteintech	SA00001-2	Goat	1:10000
Anti - Mouse IgG	Proteintech	SA00001-1	Goat	1:10000
