# Supplementary material for: PSAT1 regulates hair follicle growth and stem cell behavior in cashmere goats
Source: BMC Vet Res. 2025 Apr 16;21:277. doi: 10.1186/s12917-025-04736-6 (PMC12001598; doi:10.1186/s12917-025-04736-6)
Supplement: Supplementary file 5 — Supplementary Material 5 [file 12917_2025_4736_MOESM5_ESM.pdf]

Supplementary Table S5. Information of Antibodies Used in This Study for IF Assays.

**Antibodies**	**Source**	**Identifier**	**Host**	**Proportion**
Anti -Ki67	Proteintech	27309-1-AP	Rabbit	1:300
Anti -SOX2	Proteintech	11064-1-AP	Rabbit	1:300
Anti -OCT4	Proteintech	11263-1-AP	Rabbit	1:300
Anti -NANOG	Proteintech	14295-1-AP	Rabbit	1:100
Anti -Keratin19	Abcam	ab52625	Rabbit	1:50
Anti -PSAT1	Proteintech	10501-1-AP	Rabbit	1:300
Anti -Rabbit IgG	Proteintech	SA00003-2	Goat	1:500
Anti - Mouse IgG	Proteintech	SA00003-1	Goat	1:50
